# 4-Nitro­phenyl *N*-phenyl­carbamate

**DOI:** 10.1107/S1600536807068274

**Published:** 2008-01-09

**Authors:** Yun-Hua Xu, Fanqi Qu

**Affiliations:** a School of Science, Beijing Jiaotong University, Beijing 100044, People’s Republic of China; b College of Chemistry and Molecular Sciences, Wuhan University, Wuhan, Hubei 430072, People’s Republic of China

## Abstract

The title compound, C_13_H_10_N_2_O_4_, was synthesized as an inter­mediate for the preparation of ureas. The two aromatic rings are twisted about the central carbamate group with a C—C—N—C torsion angle of 139.6 (2)° and a C—C—O—C torsion angle of 95.9 (2)°. The mol­ecules are linked into one-dimensional chains by N—H⋯O hydrogen bonds along the *b* axis. Weak inter­actions between O atoms of the nitro groups (O⋯O = 3.012 Å) connect two adjacent chains.

## Related literature

For related literature, see: Allen *et al.* (1987 ▶); Izdebski & Pawlak (1989 ▶); Olma *et al.* (2006 ▶); Tye *et al.* (2002 ▶).
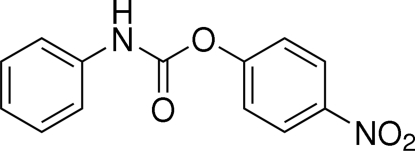


## Experimental

### 

#### Crystal data

C_13_H_10_N_2_O_4_
*M* *_r_* = 258.23
Monoclinic, 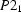

*a* = 6.0170 (2) Å
*b* = 5.0650 (1) Å
*c* = 18.8960 (5) Å
β = 92.538 (1)°
*V* = 575.31 (3) Å^3^
*Z* = 2
Mo *K*α radiation
μ = 0.11 mm^−1^
*T* = 90 (2) K
0.50 × 0.40 × 0.26 mm

#### Data collection

Nonius KappaCCD diffractometer
Absorption correction: multi-scan (*SCALEPACK*; Otwinowski & Minor, 1997 ▶) *T*_min_ = 0.946, *T*_max_ = 0.971
2630 measured reflections
1473 independent reflections
1363 reflections with *I* > 2σ(*I*)
*R*_int_ = 0.020

#### Refinement

*R*[*F* ^2^ > 2σ(*F* ^2^)] = 0.037
*wR*(*F* ^2^) = 0.104
*S* = 1.14
1473 reflections
172 parameters
1 restraint
H-atom parameters constrained
Δρ_max_ = 0.24 e Å^−3^
Δρ_min_ = −0.32 e Å^−3^

### 

Data collection: *COLLECT* (Nonius, 2002 ▶); cell refinement: *SCALEPACK* (Otwinowski & Minor, 1997 ▶); data reduction: *DENZO–SMN* (Otwinowski & Minor, 1997 ▶); program(s) used to solve structure: *SHELXS97* (Sheldrick, 2008 ▶); program(s) used to refine structure: *SHELXL97* (Sheldrick, 2008 ▶); molecular graphics: *XP* in *SHELXTL/PC* (Sheldrick, 1995 ▶); software used to prepare material for publication: *SHELXL97* and local procedures.

## Supplementary Material

Crystal structure: contains datablocks global, I. DOI: 10.1107/S1600536807068274/fl2180sup1.cif

Structure factors: contains datablocks I. DOI: 10.1107/S1600536807068274/fl2180Isup2.hkl

Additional supplementary materials:  crystallographic information; 3D view; checkCIF report

## Figures and Tables

**Table 1:** Hydrogen-bond geometry (Å, °)

*D*—H⋯*A*	*D*—H	H⋯*A*	*D*⋯*A*	*D*—H⋯*A*
N1—H1⋯O1^i^	0.88	2.05	2.903 (3)	164

Symmetry code: (i) 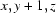.

**Fig. 1.**
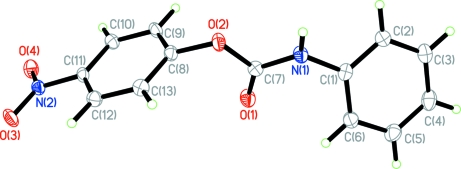
The molecular structure of (I), with displacement ellipsoids drawn at the 50% probability level (arbitrary spheres for the H atoms).

**Fig. 2.**
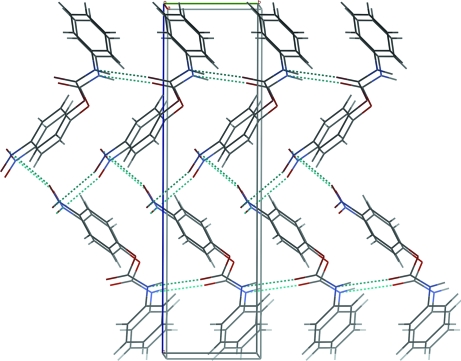
A packing diagram of (I) along a axis.

## supplementary crystallographic information

### Comment

The title compound (I), along with other 4-nitrophenyl carbamates, are important intermediates for the synthesis of ureas (Olma, *et al.* 2006; Izdebski & Pawlak, 1989). Although it has been used in organic sythesis for a long time, the crystal structure, as far as we know, is reported here for the first time.

The compound, two aromatic rings connected by a carbamate group, is not planar as would be expected. This is evidenced by the dihedral angles between the nitrophenyl ring and the carbamate, C13—C8—O2—C7=95.9 (2)°, and the benzyl ring and the carbamate, C2—C1—N1—C7=139.6 (2)°. Bond lengths and angles for (I) are within normal ranges (Allen *et al.*, 1987). The molecules form one-dimensional chains formed through N—H···O hydrogen bonds along the *b* axis (Table 1). In addition, intermolecular O···O interactions exists between nitro groups in neighboring chains (Fig. 2).

### Experimental

Aniline (1.0 g, 10.7 mmol) was added dropwise to a round-bottom flask containing 4-nitrophenyl chloroformate (2.2 g, 10.9 mmol) and pyridine (0.9 ml, 11.1 mmol) in 20 ml me thylene chloride cooled with ice water. After the solutuion was warmed to ambient temperature, the mixture was refluxed overnight with stirring. The solution was then washed with 1 N NaHCO_3_, water and brine, and then dried with anhydrous Na_2_SO_4_. After removal of the solvent, the product was recovered as a yellow solid (2.5 g, 90%) (Tye, *et al.* 2002). Colorless crystals of (I) were obtained by recrystallization from ethyl acetate.

### Refinement

H atoms were found in difference Fourier maps and subsequently placed in idealized positions with constrained C—H distances of 0.95 Å (C_Ar_H), and 0.88 Å (NH_1_). *U*_iso_(H) values were set to 1.2*U*_eq_ for all H atoms. In the absence of significant anomalous scattering effects, Friedel pairs were merged prior to refinement.

### Crystal data

**Table:**

C_13_H_10_N_2_O_4_	*F*_000_ = 268
*M**_r_* = 258.23	*D*_x_ = 1.491 Mg m^−^^3^
Monoclinic, *P*2_1_	Mo *K*α radiation λ = 0.71073 Å
Hall symbol: P2yb	Cell parameters from 1469 reflections
*a* = 6.0170 (2) Å	θ = 1–27.5º
*b* = 5.0650 (1) Å	µ = 0.11 mm^−^^1^
*c* = 18.8960 (5) Å	*T* = 90 (2) K
β = 92.538 (1)º	Block, colorless
*V* = 575.31 (3) Å^3^	0.50 × 0.40 × 0.26 mm
*Z* = 2	

### Data collection

**Table:**

Nonius KappaCCD diffractometer	1473 independent reflections
Radiation source: fine-focus sealed tube	1363 reflections with *I* > 2σ(*I*)
Monochromator: graphite	*R*_int_ = 0.020
Detector resolution: 18 pixels mm^-1^	θ_max_ = 27.5º
*T* = 90(2) K	θ_min_ = 1.1º
ω scans at fixed χ = 55°	*h* = −7→7
Absorption correction: multi-scan(SCALEPACK; Otwinowski & Minor, 1997)	*k* = −6→6
*T*_min_ = 0.946, *T*_max_ = 0.971	*l* = −24→24
2630 measured reflections	

### Refinement

**Table:**

Refinement on *F*^2^	Secondary atom site location: difference Fourier map
Least-squares matrix: full	Hydrogen site location: inferred from neighbouring sites
*R*[*F*^2^ > 2σ(*F*^2^)] = 0.038	H-atom parameters constrained
*wR*(*F*^2^) = 0.104	*w* = 1/[σ^2^(*F*_o_^2^) + (0.0582*P*)^2^ + 0.1763*P*] where *P* = (*F*_o_^2^ + 2*F*_c_^2^)/3
*S* = 1.14	(Δ/σ)_max_ = 0.009
1473 reflections	Δρ_max_ = 0.24 e Å^−^^3^
172 parameters	Δρ_min_ = −0.32 e Å^−^^3^
1 restraint	Extinction correction: none
Primary atom site location: structure-invariant direct methods	

### Special details

**Table:**

Geometry. All e.s.d.'s (except the e.s.d. in the dihedral angle between two l.s. planes) are estimated using the full covariance matrix. The cell e.s.d.'s are taken into account individually in the estimation of e.s.d.'s in distances, angles and torsion angles; correlations between e.s.d.'s in cell parameters are only used when they are defined by crystal symmetry. An approximate (isotropic) treatment of cell e.s.d.'s is used for estimating e.s.d.'s involving l.s. planes.
Refinement. Refinement of *F*^2^ against ALL reflections. The weighted *R*-factor *wR* and goodness of fit *S* are based on *F*^2^, conventional *R*-factors *R* are based on *F*, with *F* set to zero for negative *F*^2^. The threshold expression of *F*^2^ > σ(*F*^2^) is used only for calculating *R*-factors(gt) *etc*. and is not relevant to the choice of reflections for refinement. *R*-factors based on *F*^2^ are statistically about twice as large as those based on *F*, and *R*- factors based on ALL data will be even larger.

### Fractional atomic coordinates and isotropic or equivalent isotropic displacement parameters (Å^2^)

**Table:**

	*x*	*y*	*z*	*U*_iso_*/*U*_eq_	
C1	0.9665 (3)	0.7781 (5)	0.84935 (11)	0.0179 (5)	
N1	0.7888 (3)	0.8052 (4)	0.79739 (10)	0.0185 (4)	
H1	0.7443	0.9655	0.7857	0.022*	
N2	0.0763 (3)	−0.1088 (4)	0.56535 (9)	0.0180 (4)	
O1	0.7161 (3)	0.3675 (4)	0.77761 (9)	0.0227 (4)	
O2	0.5362 (3)	0.6890 (3)	0.71426 (8)	0.0196 (4)	
O3	−0.1087 (2)	−0.1712 (4)	0.58598 (9)	0.0248 (4)	
O4	0.1657 (3)	−0.2175 (4)	0.51627 (8)	0.0231 (4)	
C2	1.1431 (4)	0.9546 (5)	0.84631 (12)	0.0219 (5)	
H2	1.1414	1.0883	0.8110	0.026*	
C3	1.3226 (4)	0.9341 (6)	0.89534 (12)	0.0248 (5)	
H3	1.4432	1.0546	0.8935	0.030*	
C4	1.3255 (4)	0.7399 (6)	0.94631 (12)	0.0257 (6)	
H4	1.4489	0.7249	0.9792	0.031*	
C5	1.1480 (4)	0.5660 (6)	0.94961 (12)	0.0261 (5)	
H5	1.1498	0.4330	0.9851	0.031*	
C6	0.9670 (4)	0.5853 (6)	0.90111 (11)	0.0214 (5)	
H6	0.8451	0.4669	0.9037	0.026*	
C7	0.6852 (4)	0.5994 (5)	0.76518 (11)	0.0169 (4)	
C8	0.4214 (4)	0.4908 (5)	0.67556 (11)	0.0177 (5)	
C9	0.5166 (3)	0.3802 (5)	0.61711 (11)	0.0190 (5)	
H9	0.6578	0.4381	0.6027	0.023*	
C10	0.4012 (4)	0.1825 (5)	0.58006 (11)	0.0187 (5)	
H10	0.4625	0.1017	0.5400	0.022*	
C11	0.1954 (3)	0.1055 (5)	0.60271 (11)	0.0161 (4)	
C12	0.0975 (4)	0.2213 (5)	0.65993 (11)	0.0189 (5)	
H12	−0.0457	0.1672	0.6735	0.023*	
C13	0.2135 (4)	0.4187 (5)	0.69708 (11)	0.0198 (5)	
H13	0.1508	0.5024	0.7365	0.024*	

### Atomic displacement parameters (Å^2^)

**Table:**

	*U*^11^	*U*^22^	*U*^33^	*U*^12^	*U*^13^	*U*^23^
C1	0.0170 (10)	0.0182 (11)	0.0183 (9)	0.0038 (10)	−0.0015 (7)	−0.0028 (9)
N1	0.0191 (9)	0.0118 (9)	0.0241 (9)	0.0013 (8)	−0.0045 (7)	−0.0005 (8)
N2	0.0152 (9)	0.0161 (10)	0.0224 (8)	−0.0008 (8)	−0.0022 (7)	0.0029 (9)
O1	0.0238 (8)	0.0150 (9)	0.0286 (8)	0.0000 (7)	−0.0060 (6)	0.0001 (7)
O2	0.0188 (8)	0.0154 (8)	0.0240 (7)	−0.0009 (7)	−0.0058 (6)	−0.0014 (7)
O3	0.0153 (7)	0.0257 (10)	0.0334 (8)	−0.0076 (8)	0.0006 (6)	0.0016 (8)
O4	0.0207 (8)	0.0215 (9)	0.0269 (8)	0.0002 (8)	−0.0016 (6)	−0.0052 (8)
C2	0.0230 (11)	0.0208 (13)	0.0218 (10)	−0.0031 (10)	−0.0008 (8)	0.0009 (10)
C3	0.0205 (11)	0.0267 (14)	0.0269 (11)	−0.0036 (10)	−0.0020 (8)	−0.0045 (10)
C4	0.0218 (11)	0.0264 (14)	0.0280 (11)	0.0036 (11)	−0.0076 (9)	−0.0041 (11)
C5	0.0303 (13)	0.0235 (13)	0.0238 (10)	0.0022 (12)	−0.0061 (9)	0.0027 (11)
C6	0.0215 (11)	0.0204 (12)	0.0221 (10)	−0.0020 (11)	−0.0017 (8)	0.0002 (10)
C7	0.0163 (10)	0.0155 (11)	0.0190 (9)	0.0017 (9)	0.0008 (7)	−0.0003 (9)
C8	0.0188 (10)	0.0130 (11)	0.0209 (10)	−0.0012 (10)	−0.0049 (8)	0.0010 (9)
C9	0.0127 (9)	0.0196 (12)	0.0244 (10)	−0.0032 (10)	−0.0012 (8)	0.0019 (10)
C10	0.0151 (10)	0.0198 (11)	0.0212 (9)	−0.0010 (9)	0.0013 (8)	−0.0002 (9)
C11	0.0133 (10)	0.0146 (10)	0.0200 (9)	−0.0004 (9)	−0.0039 (7)	0.0013 (9)
C12	0.0151 (10)	0.0201 (13)	0.0215 (10)	0.0008 (9)	0.0004 (8)	0.0019 (9)
C13	0.0191 (10)	0.0201 (12)	0.0203 (9)	0.0011 (10)	0.0012 (8)	−0.0004 (9)

### Geometric parameters (Å, °)

**Table:**

C1—C6	1.382 (3)	C4—C5	1.388 (4)
C1—C2	1.392 (4)	C4—H4	0.9500
C1—N1	1.426 (3)	C5—C6	1.396 (3)
N1—C7	1.346 (3)	C5—H5	0.9500
N1—H1	0.8800	C6—H6	0.9500
N2—O4	1.224 (3)	C8—C13	1.381 (3)
N2—O3	1.237 (2)	C8—C9	1.385 (3)
N2—C11	1.465 (3)	C9—C10	1.390 (3)
O1—C7	1.210 (3)	C9—H9	0.9500
O2—C7	1.364 (3)	C10—C11	1.384 (3)
O2—C8	1.405 (3)	C10—H10	0.9500
C2—C3	1.395 (3)	C11—C12	1.385 (3)
C2—H2	0.9500	C12—C13	1.391 (3)
C3—C4	1.376 (4)	C12—H12	0.9500
C3—H3	0.9500	C13—H13	0.9500
			
C6—C1—C2	120.4 (2)	C1—C6—H6	120.3
C6—C1—N1	122.2 (2)	C5—C6—H6	120.3
C2—C1—N1	117.4 (2)	O1—C7—N1	126.8 (2)
C7—N1—C1	123.7 (2)	O1—C7—O2	123.4 (2)
C7—N1—H1	118.1	N1—C7—O2	109.8 (2)
C1—N1—H1	118.1	C13—C8—C9	122.7 (2)
O4—N2—O3	123.8 (2)	C13—C8—O2	117.8 (2)
O4—N2—C11	118.45 (18)	C9—C8—O2	119.5 (2)
O3—N2—C11	117.76 (19)	C8—C9—C10	118.6 (2)
C7—O2—C8	114.97 (19)	C8—C9—H9	120.7
C3—C2—C1	119.7 (2)	C10—C9—H9	120.7
C3—C2—H2	120.2	C11—C10—C9	118.8 (2)
C1—C2—H2	120.2	C11—C10—H10	120.6
C4—C3—C2	120.2 (2)	C9—C10—H10	120.6
C4—C3—H3	119.9	C10—C11—C12	122.7 (2)
C2—C3—H3	119.9	C10—C11—N2	119.0 (2)
C3—C4—C5	120.0 (2)	C12—C11—N2	118.30 (19)
C3—C4—H4	120.0	C11—C12—C13	118.5 (2)
C5—C4—H4	120.0	C11—C12—H12	120.8
C4—C5—C6	120.4 (2)	C13—C12—H12	120.8
C4—C5—H5	119.8	C8—C13—C12	118.8 (2)
C6—C5—H5	119.8	C8—C13—H13	120.6
C1—C6—C5	119.4 (2)	C12—C13—H13	120.6
			
C6—C1—N1—C7	−40.3 (3)	C7—O2—C8—C9	−85.5 (3)
C2—C1—N1—C7	139.6 (2)	C13—C8—C9—C10	−2.2 (4)
C6—C1—C2—C3	0.8 (4)	O2—C8—C9—C10	179.3 (2)
N1—C1—C2—C3	−179.1 (2)	C8—C9—C10—C11	0.5 (3)
C1—C2—C3—C4	0.2 (4)	C9—C10—C11—C12	1.4 (4)
C2—C3—C4—C5	−0.9 (4)	C9—C10—C11—N2	−178.0 (2)
C3—C4—C5—C6	0.5 (4)	O4—N2—C11—C10	1.8 (3)
C2—C1—C6—C5	−1.1 (4)	O3—N2—C11—C10	−179.0 (2)
N1—C1—C6—C5	178.8 (2)	O4—N2—C11—C12	−177.6 (2)
C4—C5—C6—C1	0.5 (4)	O3—N2—C11—C12	1.6 (3)
C1—N1—C7—O1	4.5 (4)	C10—C11—C12—C13	−1.6 (4)
C1—N1—C7—O2	−174.95 (18)	N2—C11—C12—C13	177.8 (2)
C8—O2—C7—O1	−1.1 (3)	C9—C8—C13—C12	2.0 (4)
C8—O2—C7—N1	178.43 (18)	O2—C8—C13—C12	−179.5 (2)
C7—O2—C8—C13	95.9 (2)	C11—C12—C13—C8	−0.1 (3)

### Hydrogen-bond geometry (Å, °)

**Table:**

*D*—H···*A*	*D*—H	H···*A*	*D*···*A*	*D*—H···*A*
N1—H1···O1^i^	0.88	2.05	2.903 (3)	164

Symmetry codes: (i) *x*, *y*+1, *z*.
